# Co-Overexpression of GRK5/ACTC1 Correlates With the Clinical Parameters and Poor Prognosis of Epithelial Ovarian Cancer

**DOI:** 10.3389/fmolb.2021.785922

**Published:** 2022-02-09

**Authors:** Longyang Liu, Jin Lv, Zhongqiu Lin, Yingxia Ning, Jing Li, Ping Liu, Chunlin Chen

**Affiliations:** ^1^ Department of Gynecology and Obstetrics, Nanfang Hospital, Southern Medical University, Guangzhou, China; ^2^ Cancer Center, Integrated Hospital of Traditional Chinese Medicine, Southern Medical University, Guangzhou, China; ^3^ Department of Obstetrics and Gynecology, Longgang Central Hospital of Shenzhen City, Shenzhen, China; ^4^ Department of Gynecology Oncology, The Memorial Hospital of Sun Yat-sen University, Guangzhou, China; ^5^ Department of Gynecology and Obstetrics, The First Affiliated Hospital of Guangzhou Medical University, Guangzhou, China

**Keywords:** GRK5, ACTC1, epithelial ovarian cancer, prognosis, expression

## Abstract

**Background:** The prognosis of epithelial ovarian cancer (EOC) is poor, and the present prognostic predictors of EOC are neither sensitive nor specific.

**Objective:** The aim of this study was to search the prognostic biomarkers of EOC and to investigate the expression of G protein-coupled receptor kinase 5 (GRK5) and actin alpha cardiac muscle 1 (ACTC1) in EOC tissues (both paraffin-embedded and fresh-frozen tissues) and to explore their association with clinicopathological parameters and prognostic value in patients with EOC.

**Methods:** A total of 172 paraffin-embedded cancer tissues of EOC patients diagnosed and operated at the memorial hospital of Sun Yat-sen University between December 2009 and March 2017 and 41 paratumor tissues were collected and the expression of GRK5 and ACTC1 was examined using immunohistochemistry. Furthermore, 16 fresh-frozen EOC tissues and their matched paratumor tissues were collected from the Integrated Hospital of Traditional Chinese Medicine, Southern Medical University, between August 2013 and November 2019 and subjected to reverse-transcription quantitative PCR analysis to detect the mRNA expression of GRK5 and ACTC1.

**Results:** The expression of GRK5 and ACTC1 was both higher in cancer tissues than in paratumor tissues. GRK5 expression was positively correlated with ACTC1 expression. In addition, GRK5, ACTC1, and GRK5/ACTC1 expression was associated with the recurrence-free survival and overall survival of EOC patients. Furthermore, multivariate logistic regression analysis indicated that GRK5+/ACTC1+ co-expression, intestinal metastasis, postoperative chemotherapy, platinum resistance, and hyperthermic intraperitoneal chemotherapy were independent prognostic factors of EOC.

**Conclusion:** GRK5 and ACTC1 are both upregulated in EOC compared with those in paratumor tissues. The co-expression of GRK5+/ACTC1+ rather than GRK5 or ACTC1 is an independent prognostic biomarker of EOC.

## Introduction

Ovarian cancer (OC) is the main cause of mortality in female reproductive malignant cancers in China (Chen et al., 2016), and it is the second most common cause of gynecologic cancer-related death in women worldwide (Lheureux et al., 2019). Globally, there are 239,000 new cases and 152,000 deaths every year, making OC the seventh most common cancer and the second most common cause of gynecologic cancer-related mortality (Lheureux et al., 2019). The most common type of OC is epithelial ovarian cancer (EOC). Cytoreduction and combination chemotherapies were performed to treat OC, but the prognosis remains poor (Liu et al., 2019a; Yao et al., 2019; Liu et al., 2020). Recently, tumor biomarkers have been used to monitor the progression and predict the prognosis of EOC, but these biomarkers are not very accurate (Liu et al., 2019a; Yao et al., 2019; Liu et al., 2020).

In our previous study, we found that non-muscle myosin heavy chain B (MYH10) is an independent prognostic biomarker of EOC (in print). Furthermore, we used the Biogrid website to predict the candidate interacting proteins of MYH10, and we found some candidate proteins that may closely correlate with MYH10, such as MYL9 (Deng et al., 2021), MACF1 (Liu et al., 2021), MYH9 (Liu et al., 2019b), and so on. In the further study, we found that the co-expression of GRK5 (G protein-coupled receptor kinase 5) and ACTC1 (actin alpha cardiac muscle 1) is indeed independent prognostic biomarkers, which indicates their important role in EOC. GRK5 is one of the G protein-coupled receptor kinase (GRK) family members (Gambardella et al., 2016; Jiang et al., 2018; Zhao et al., 2019a; Lagman et al., 2019; Sommer et al., 2019), which is a candidate interacting protein of MYH10. GRK5 can regulate GPCR signaling, which correlates with various diseases like cardiac dysfunction, diabetes, hypertension, Alzheimer’s disease, and cancers (Kim et al., 2012; Komolov et al., 2017; Jiang et al., 2018; Alshabi et al., 2019; Zhao et al., 2019b). GRK5 functions as oncogenes in glioblastoma (GBM) (Yang et al., 2018), prostate (Kim et al., 2012), pancreas (Tseng and Zhang, 2000), non-small-cell lung (Jiang et al., 2018), and breast (Sommer et al., 2019) cancers. However, to the best of our knowledge, the role of GRK5 in EOC has not been reported.

Similar to GRK5, ACTC1 is also a cardiac-related gene (Kondrashov et al., 2018), and both of them are the candidate interacting proteins of MYH10. ACTC1 encodes cardiac actin, and a mutation at c.G301A causes hypertrophic cardiomyopathy and, in some cases, sudden cardiac death (Frank et al., 2019). Recently, some reports (Kim et al., 2019; da Rocha et al., 2019; Wanibuchi et al., 2018; Cheung et al., 2017; Li et al., 2017; Ohtaki et al., 2017; Zaravinos et al., 2011) have demonstrated that ACTC1 plays an important role in human colon cancer, oral squamous cell carcinoma, GBM, and so on. However, the role of ACTC1 in EOC has not been reported, and the relationship of GRK5 and ACTC1 and EOC has not been explored yet.

The present study identified that GRK5 and ACTC1 were both upregulated in EOC. More importantly, GRK5+/ACTC1+ co-expression was an independent prognostic factor. The GRK5+/ACTC1+ co-expression could predict the development, metastasis, and prognosis of EOC.

## Materials and Methods

### Paraffin-Embedded Tissue Sections

Between December 2009 and March 2017, a total of 172 paraffin-embedded EOC tissues and 41 matched paraffin-embedded paratumor tissues (the distance away from the margin of the cancer tissue is more than 1.0 cm) that had been pathologically confirmed at the memorial hospital of Sun Yat-sen University were collected for the present study. The survival duration was calculated from the date of surgery to November 1, 2018 (last follow-up). The approval of the present study was obtained from the Ethics Committee of the memorial hospital of Sun Yat-sen University. All of the patients provided written informed consent prior to the operation.

### Fresh Tissue Specimens

Between August 2013 and June 2019, 16 fresh EOC tissues and their matched fresh paratumor tissues were collected from the Integrated Hospital of Traditional Chinese Medicine, Southern Medical University, at the time of diagnosis after surgery. All fresh samples were immediately preserved in liquid nitrogen. Approval was obtained from the Ethics Committee of the Integrated Hospital of Traditional Chinese Medicine, Southern Medical University. All of the patients provided written informed consent prior to surgery.

### Immunohistochemistry

The expression of GRK5 and ACTC1 in paraffin-embedded EOC and paired paratumor tissues was detected by IHC staining. First, 4-μm paraffin-embedded sections were baked at 65°C for 2 h, deparaffinized with xylene, and rehydrated; high tension was used for antigen retrieval, and the specimens were treated with 3% hydrogen peroxide in methanol, followed by incubation with 1% bovine serum albumin to block non-specific binding. Subsequently, the samples were incubated with anti-rabbit GRK5 polyclonal (1:150 dilution; Cat. 17032-1-AP; Proteintech) or ACTC1 antibodies (1:200 dilution; Cat. 66125-1-IG; Proteintech) at 4°C overnight. Next, the samples were treated with secondary antibody (OriGene, Rockville, MD, USA) and then incubated with streptavidin horseradish peroxidase complex (OriGene, Rockville, MD, USA), immersed in 3-amino-9-ethyl carbazole. The sections were then counterstained with 10% Mayer’s hematoxylin, dehydrated, and mounted in Crystal Mount. Two pathologists evaluated the score of immunostaining for each section. The score was based on the proportion of positively stained cancer cells and the staining intensity. The percentage was scored as follows: samples with <10% positive cancer cells were scored as 0; 10–50% were scored as 1, 50–75% were scored as 2, and >75% were scored as 3. Furthermore, the tissues were classified into four grades based on staining intensity: 0 indicated no staining, 1 indicated weak staining, 2 indicated moderate staining, and 3 indicated strong staining. The staining index (0–9) was calculated as the product of the proportion of positive cells multiplied by the staining intensity score. The best cutoff value was defined as follows: a staining score of ≥6 was considered to indicate high GRK5 or ATCT1 protein expression (also called GRK5+ or ACTC1+), and a staining score of ≤5 indicated low GRK5 or ATCT1 protein expression (also called GRK5- or ACTC1-) (Fu et al., 2017; Zhen et al., 2017; Zhao et al., 2018; Liang et al., 2019; Zou et al., 2019).

### Real-Time Quantitative Polymerase Chain Reaction

The total RNA was extracted from the EOC tissues and paratumor tissues by using TRIzol (Takara Bio, Inc., Shiga, Japan). GAPDH mRNA was detected as the internal control (Liu et al., 2020). The expression levels of each matched fresh paratumor tissue sample were set as the control group (the expression levels of MYL9 in all of the paratumor tissues were 1.00 ± 0.00), and the relative expression is 2^-∆∆Ct^. The thermocycling conditions (Zhao et al., 2016; Liu et al., 2019c; Li et al., 2019; Lin et al., 2019; Xiao et al., 2019) were 95°C for 10 min to activate DNA polymerase, followed by 45 cycles of 95°C for 15 s, 60°C for 15 s, and 72°C for 10 s. The specificity of amplification products was confirmed by melting curve analysis. Independent experiments were performed in triplicate. The specific primer sequences were as follows: GRK5 forward, 5′-CCT​CCG​AAG​GAC​CAT​AGA​CA-3′ and reverse, 5′-GAC​TGG​GGA​CTT​TGG​AGT​GA-3’; ACTC1 forward, 5′-GGT​GAT​GAA​GCC​CAG​AGC​AA-3′ and reverse, 5′-GTG​GTG​ACA​AAG​GAG​TAG​CC-3’; GAPDH forward, 5′-CCA​TCT​TCC​AGG​AGC​GAG​AT-3′ and reverse, 5′-TGC​TGA​TGA​TCT​TGA​GGC​TG-3’.

### Statistical Analysis

All data analyses were performed using the statistical software package SPSS 21.0 (IBM Corp.) and GraphPad Prism 7 (GraphPad Software, Inc.). The mRNA expression of GRK5 or ACTC1 was expressed as the mean ± standard deviation. A two-tailed Student’s t-test was used for comparisons between two independent groups (the expression of GRK5 or ACTC1 in paratumor tissues as the control group). The chi-square test or Fisher’s exact test was used to analyze the association among GRK5 or ACTC1 or GRK5/ACTC1 co-expression (including GRK5+/ACTC1+, GRK5-/ACTC1+, GRK5+/ACTC1-, and GRK5-/ACTC1-) and clinicopathological parameters. Furthermore, the recurrence-free survival (RFS) and overall survival (OS) were analyzed by Kaplan–Meier analysis, and the differences were assessed using the log-rank test. Cox’s proportional hazards regression model was used for univariate and multivariate analysis. Spearman or Pearson correlation was used for the correlation between GRK5 and ACTC1 expression. *p* < 0.05 was considered to indicate statistical significance.

## Results

### GRK5 and ACTC1 mRNA Were Both Upregulated in Fresh Epithelial Ovarian Cancer Tissues Compared With That in Paratumor Tissues

**FIGURE 1:**
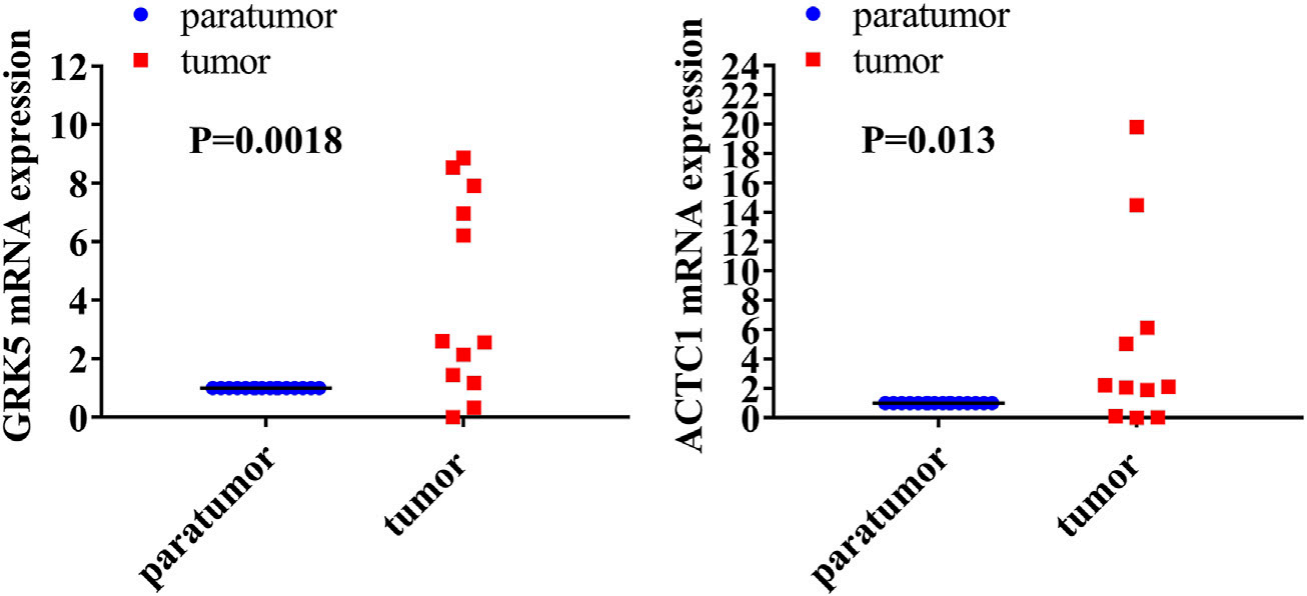
Both GRK5 and ACTC1 were significantly upregulated in EOC tissues compared with that in paratumor tissues using RT-qPCR analysis.

Reverse-transcription quantitative PCR (RT-qPCR) analysis was performed to detect the mRNA expression levels of both GRK5 and ACTC1 in 16 fresh EOC tissues and matched paratumor tissues (Figure 1). The expression of GRK5 or ACTC1 in all of the matched paratumor tissues was set as 1.00 ± 0.00, and the expression in each of the tumor tissues was compared with that in the matched paratumor tissues. The results indicated that the mean expression of GRK5 and ACTC1 in the 16 fresh EOC tissues was 21.590 and 245.600, respectively. There was a significant difference between EOC tissues and paratumor tissues (*p* = 0.0018; *p* = 0.013; Table 1 and Figure 1).

**TABLE 1:** Both GRK5 and ACTC1 were significantly upregulated in EOC tissues compared with that in paratumor tissues.

Group	GRK5 mRNA expression	ACTC1 mRNA expression
All of paratumor tissues (total of 16 cases)	1.00 ± 0.00	1.00 ± 0.00
Patient 1	6.218 ± 4.482	14.480 ± 5.654
Patient 2	6.963 ± 3.018	5.057 ± 3.370
Patient 3	8.547 ± 2.435	166.100 ± 105.700
Patient 4	8.865 ± 1.137	833.800 ± 584.800
Patient 5	2.610 ± 0.800	0.115 ± 0.075
Patient 6	2.149 ± 0.737	0.016 ± 0.004
Patient 7	1.442 ± 0.489	2.229 ± 0.837
Patient 8	29.440 ± 31.740	53.560 ± 20.970
Patient 9	0.002 ± 0.145	0.029 ± 0.010
Patient 10	96.510 ± 58.720	246.700 ± 29.080
Patient 11	2.564 ± 2.011	1.885 ± 0.405
Patient 12	0.326 ± 0.116	2.079 ± 0.790
Patient 13	1.173 ± 0.165	6.146 ± 1.582
Patient 14	7.917 ± 5.036	2.116 ± 0.705
Patient 15	22.360 ± 9.835	19.800 ± 2.400
Patient 16	148.300 ± 30.390	2,575.000 ± 312.400
Expression of all patients	21.590 ± 43.210**	245.600 ± 656.600*
*p*	**0.0018**	0.013

ACTC1, actin alpha cardiac muscle 1; GRK5, G protein-coupled receptor kinase 5. Bold value indicates the significant differences.

### GRK5 and ACTC1 Expression Were Both Assessed in Paraffin-Embedded Epithelial Ovarian Cancer Tissues and Paratumor Tissues by Immunohistochemistry

**FIGURE 2:**
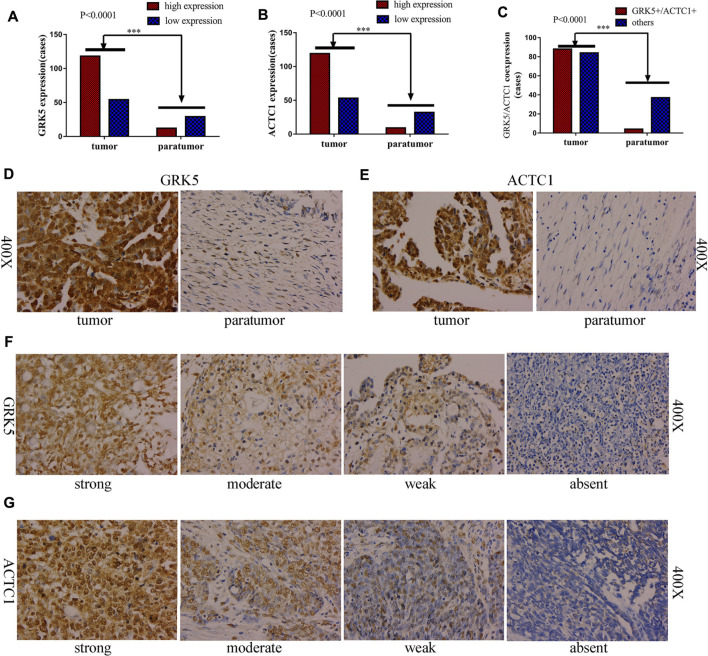
GRK5 and ACTC1 were both upregulated in paraffin-embedded EOC. **(A)** GRK5 expression in EOC tissues and paratumor tissues (*p* < 0.0001); **(B)** ACTC1 expression in EOC tissues and paratumor tissues (*p* < 0.0001); **(C)** GRK5/ACTC1 co-expression in EOC tissues and paratumor tissues (*p* < 0.0001); **(D)** Comparison of EOC/paratumor tissues immunohistochemically stained for GRK5 (400X); **(E)** Comparison of EOC/paratumor tissues immunohistochemically stained for ACTC1 (400X); **(F)** the staining of GRK5 expression in EOC tissues (400X); **(G)** the staining of ACTC1 expression in EOC tissues (400X).

To further determine whether GRK5 or ACTC1 protein is upregulated in EOC, 172 paraffin-embedded EOC tissues and 41 matched paratumor tissues were subjected to the IHC analysis of GRK5 and ACTC1 expression. The results indicated that 54/172 (31.40%, GRK5) and 53/172 (30.81%, ACTC1) of the cancer samples had low/absent staining (rated as low expression) and 118/172 (68.60%, GRK5) and 119/172 (69.19%, ACTC1) had moderate/strong staining (rated as high expression), while the IHC analysis of the 41 paratumor tissues indicated that 29/41 (70.73%, GRK5) and 32/41 (78.05%, ACTC1) of the paratumor samples had low/absent staining and 12/41 (29.27%, GRK5) and 9/41 (21.95%, ACTC1) had moderate/strong staining. Moreover, the results indicated that 88/172 (51.16%, GRK5+/ACTC1+) of the cancer samples had moderate/strong staining and 84/172 (48.84%, others) had low/absent staining, while the IHC analysis of the 41 paratumor tissues indicated that 37/41 (90.24%, others) and low/absent staining and 4/41 (9.76%, GRK5+/ACTC1+) had moderate/strong staining. There was a significant difference on the GRK5, ACTC1, and GRK5/ACTC1 expression between the cancer and paratumor tissues (*p* < 0.0001, *p* < 0.0001, *p* < 0.0001; Figures 2A–C). Furthermore, it was observed that GRK5 and ACTC1 proteins were both located in the nucleus (Figures 2D–G).

### GRK5 or ACTC1 or GRK5/ACTC1 Co-expression Was Associated With Recurrence-Free Survival and Overall Survival of Epithelial Ovarian Cancer Patients

**FIGURE 3:**
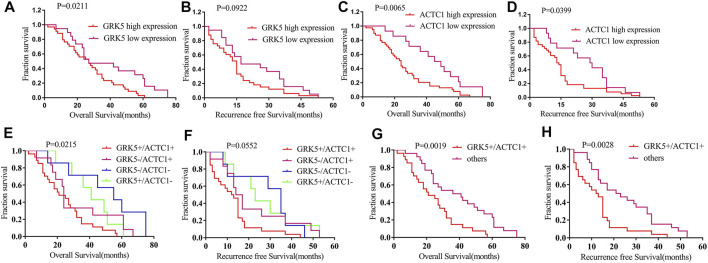
Kaplan–Meier survival of RFS and OS among GRK5 and ACTC1 and co-expression of GRK5/ACTC1 in EOC patients: **(A)** Kaplan–Meier survival of OS between GRK5 expression and EOC patients; **(B)** Kaplan–Meier survival of RFS between GRK5 expression and EOC patients; **(C)** Kaplan–Meier survival of OS between ACTC1 expression and EOC patients; **(D)** Kaplan–Meier survival of RFS between ACTC1 expression and EOC patients; **(E,G)** Kaplan–Meier survival of OS between GRK5/ACTC1 co-expression and EOC patients; **(F,H)** Kaplan–Meier survival of RFS between GRK5/ACTC1 co-expression and EOC patients.

In this study, patients with GRK5+ exhibited a median OS time of 27 months, while patients with GRK5- exhibited a median OS time of 27 months (HR = 1.81, 95% CI: 1.056–3.101). Patients with GRK5+ exhibited a median RFS time of only 15 months, while patients with GRK5- exhibited a median RFS time of 17 months (HR = 1.555, 95% CI: 0.9023–2.681). Moreover, patients with ACTC1+ exhibited a median OS time of only 24 months, while patients with ACTC1- exhibited a median OS time of 47 months (HR = 2.159, 95% CI: 1.255–3.716). Patients with ACTC1+ exhibited a median RFS time of only 14 months, while patients with ACTC1- exhibited a median RFS time of 29.5 months (HR = 1.784, 95% CI: 1.024–3.11). In addition, patients with GRK5+/ACTC1+ co-expression exhibited a median OS time of only 21.0 months, while patients with others co-expression exhibited a mean OS time of 38.5 months (HR = 2.17, 95% CI: 1.22–3.858). Patients with GRK5+/ACTC1+ co-expression exhibited a median RFS time of only 12.5 months, while patients with other co-expressions exhibited a mean RFS time of 22 months (HR = 2.092, 95% CI: 1.17–3.739). Kaplan–Meier survival analysis demonstrated that there was a statistical significance on the OS and RFS between GRK5+/ACTC1+ and others co-expression (*p* = 0.0019 and *p* = 0.0028, respectively), and there was also a statistical significance on the OS and RFS between ACTC1+ and ACTC1- (*p* = 0.0065 and *p* = 0.0399, respectively). However, there was a significant difference between the GRK5+ and GRK5- on OS (*p* = 0.0211), but there was no significance on the RFS between GRK5+ and GRK5- (*p* = 0.0922) (Figure 3). A survival analysis showed that the cumulative OS and RFS rates of EOC patients increased with the increase in GRK5+/ACTC1+ co-expression (Figure 3).

### GRK5 and ACTC1 and GRK5/ACTC1 Co-expression Were Associated With the Clinicopathological Parameters of Epithelial Ovarian Cancer Patients

Subsequently, we evaluated their correlation with the clinicopathological parameters of EOC patients. The results of using χ2 or Fisher’s exact test showed that there were significant relationships between GRK5 expression and clinicopathological parameters of EOC, such as the following factors: FIGO (International Federation of Gynecology and Obstetrics) stage, intraperitoneal metastasis, intestinal metastasis, differentiation grade, ascites with tumor cells, and so on (Table 2). There were significant relationships between ACTC1 expression and the clinicopathological parameters of EOC, such as the following factors: FIGO stage, lymph node metastasis, intraperitoneal metastasis, intestinal metastasis, vital status, and so on (Table 2). At last, we used χ2 or Fisher’s exact test to explore the relationship between GRK5/ACTC1 co-expression and the clinicopathological parameters of EOC, and we found that there were significant differences in the following factors: histology, FIGO stage, lymph node metastasis, intraperitoneal metastasis, intestinal metastasis, vital status, differentiation grade, ascites with tumor cells, and so on (Table 2).

**TABLE 2:** GRK5, ACTC1 and GRK5/ACTC1 co-expression in association with clinical parameters of EOC.

Parameters		Total	GRK5			*ACTC1*			Co-expression of GRK5/ACTC1		
			Low	High	*p*-value (χ2 or Fisher’s exact test)	Low	High	*p*-value (χ2 or Fisher’s exact test)	GRK5+/ACTC1+	Others	*p*-value (χ2 or Fisher’s exact test)
Age (years)	≤50	75	27	48	0.2525	25	50	0.5292	33	42	0.0984
	>50	97	27	70		28	69		55	42	
Histology	Serous	110	29	81	0.0580	29	81	0.0729	65	45	**0.0095**
	Mucoid	10	6	4		5	5		2	8	
	Endometrial	22	10	12		7	15		7	15	
	Clear cell	12	5	7		7	5		4	8	
FIGO stage	I/II	48	24	24	**0.0011**	25	23	**0.0002**	11	37	**<0.0001**
	III/IV	124	30	94		28	96		77	47	
Lymph node metastasis	No	47	23	24	0.0832	24	23	**0.0266**	12	35	**0.0062**
	Yes	28	8	20		7	21		16	12	
Intraperitoneal metastasis	No	57	25	32	**0.0131**	29	28	**<0.0001**	16	41	**<0.0001**
	Yes	115	29	86		24	91		72	43	
Intestinal metastasis	No	93	44	49	**<0.0001**	39	54	**0.0006**	29	64	**<0.0001**
	Yes	79	10	69		14	65		59	20	
Vital status	Alive	60	24	36	0.6502	28	32	**0.0262**	19	41	**0.0374**
	Dead	53	19	34		14	39		27	26	
Intraperitoneal recurrence	No	122	38	84	0.8558	41	81	0.3497	60	62	0.5503
	Yes	46	15	31		12	34		25	21	
Distant recurrence	No	140	45	95	0.7105	47	93	0.2069	69	71	0.4478
	Yes	28	8	20		6	22		16	12	
Differentiation grade	G1/G2	58	26	32	**0.0029**	21	37	0.1418	22	36	**0.0046**
	G3	103	23	80		26	77		63	40	
Platinum resistance	No	164	50	114	0.1784	50	114	0.6438	84	80	0.6782
	Yes	5	3	2		2	3		2	3	
Ascites with tumor cells	No	45	22	23	**0.0077**	16	29	0.5082	16	29	**0.0153**
	Yes	35	7	28		10	25		22	13	
CA125 (U/ml)	≤35	22	7	15	0.8794	4	18	0.3150	13	9	0.4464
	>35	139	42	97		44	95		70	69	
**CA72-4** (U/ml)	≤7	69	23	46	0.5078	22	47	0.6315	33	36	0.3135
	>7	71	20	51		20	51		40	31	
CA153 (U/ml)	≤25	11	5	6	0.3095	3	8	>0.9999	6	5	0.9267
	>25	41	12	29		10	31		23	18	
AFP (U/ml)	≤25	130	40	90	>0.9999	40	90	>0.9999	66	64	>0.9999
	>25	1	0	1		0	1		1	0	
CEA (U/ml)	≤5	117	35	82	0.8534	34	83	0.1893	61	56	0.5433
	>5	18	5	13		8	10		8	10	
HE4 (U/ml)	≤140	30	8	22	0.6835	9	21	0.5101	17	13	0.2746
	>140	65	20	45		24	41		29	36	

ACTC1, actin alpha cardiac muscle 1; FIGO, International Federation of Gynecology and Obstetrics; GRK5, G protein-coupled receptor kinase 5. Bold values indicate the significant differences.

### Correlation Between GRK5 and ACTC1 Expression

To explore the relationship between GRK5 and ACTC1, Spearman correlation and χ2 tests were used for analysis, and the results showed that there is a statistical significance between them (*p* = 0.0236) (Table 3).

**TABLE 3:** Correlation between GRK5 and ACTC1 expression.

GRK5	ACTC1		Spearman’s R	**χ2**	*p*
	High	Low			
high	88	30	0.173	5.122	0.0236
Low	31	23			

ACTC1, actin alpha cardiac muscle 1; GRK5, G protein-coupled receptor kinase 5

### GRK5 mRNA Was Positively Correlated With ACTC1 mRNA

**FIGURE 4:**
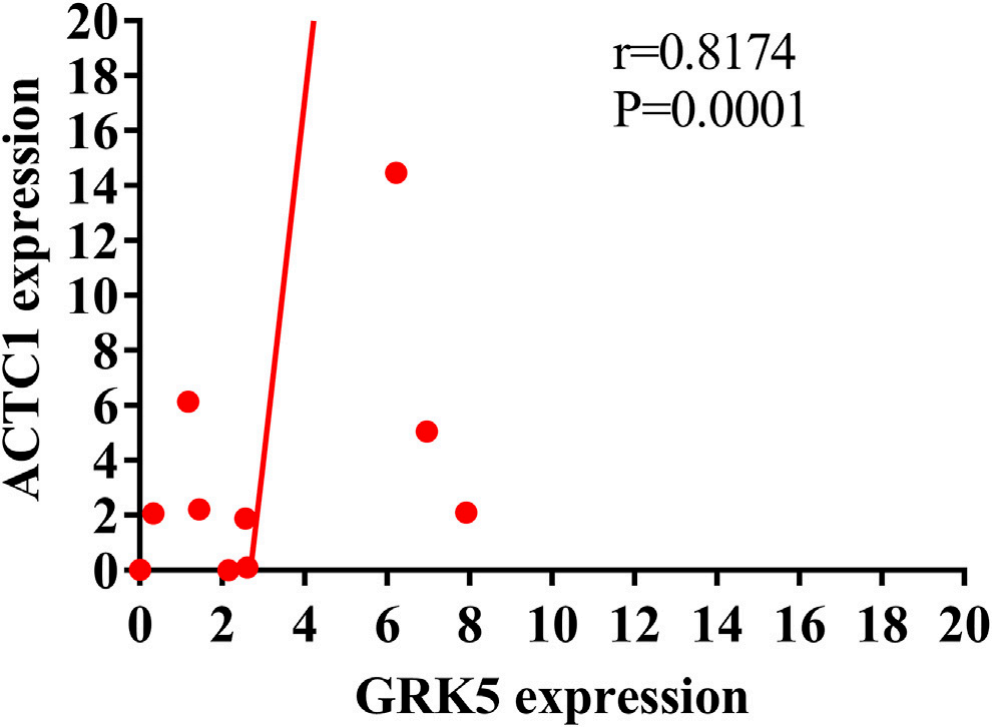
Pearson correlation analysis showing the positive correlation of GRK5 mRNA and ACTC1 mRNA.

Further, we assessed the correlation between GRK5 and ACTC1 mRNA expression. Using Pearson correlation analysis, we found that GRK5 was positively correlated with ACTC1 (*r* = 0.8174, *p* = 0.0001) (Figure 4).

### Co-Expression of GRK5+/ACTC1+ Was a Useful Independent Prognostic Predictor of Epithelial Ovarian Cancer

Furthermore, we also assessed the prognostic value of GRK5, ACTC1, and GRK5/ACTC1 co-expression in EOC patients. In a univariate Cox model analysis, GRK5, ACTC1, and GRK5/ACTC1 co-expression; intestinal metastasis; postoperative chemotherapy; platinum resistance; and hyperthermic intraperitoneal chemotherapy (HIPEC) were significant prognostic factors (Table 4). Moreover, in a multivariate Cox regression model, we found that GRK5+/ACTC1+ co-expression, intestinal metastasis, postoperative chemotherapy, platinum resistance, and HIPEC were indeed independent prognostic factors of EOC (Table 4), but GRK5 expression and ACTC1 expression were no longer significant.

**TABLE 4:** Cox regression univariate and multivariate analyses of prognostic factors in EOC.

Variable	Univariate analysis				Multivariate analysis		
	Number of patients	*p*	Exp(B)/OR	95% confidence interval	*p*	Hazard ratios	95% confidence interval
GRK5		0.026	1.427	1.044–1.951	0.967	-	-
High expression	118						
Low expression	54						
ACTC1		0.010	1.729	1.239–2.411	0.482	-	-
High expression	119						
Low expression	53						
**Co-expression of GRK5/ACTC1**		0.003	0.399	0.218–0.730	**0.011**	**0.425**	0.220–0.821
Others	84						
GRK5+/ACTC1+	88						
Intestinal metastasis		0.016	2.110	1.147–3.882	**0.010**	**2.515**	1.249–5.063
Yes	79						
No	93						
Postoperative chemotherapy		0.035	0.192	0.041–0.891	**0.006**	**0.095**	0.018–0.501
Yes	164						
No	6						
Platinum resistance		0.040	0.285	0.086–0.945	**0.001**	**0.021**	0.069–0.802
No	164						
Yes	5						
**HIPEC**		0.014	16.913	1.759–162.606	**0.021**	**84.504**	6.866–1,040.019
No	152						
Yes	19						

ACTC1, actin alpha cardiac muscle 1; GRK5, G protein-coupled receptor kinase 5; HIPEC, hyperthermic intraperitoneal chemotherapy. Bold values indicate the significant differences.

## Discussions

In our previous study, we found that MYH10 is an independent prognostic biomarker of EOC (in print). Furthermore, we used the Biogrid website to predict the candidate interacting proteins of MYH10, and we found some candidate proteins that may closely correlate with MYH10, such as MYL9 (Deng et al., 2021), MACF1 (Liu et al., 2021), MYH9 (Liu et al., 2019b), and so on. In the further study, we found that the co-expression of GRK5 and ACTC1 is indeed independent prognostic biomarkers, which indicates their important role in EOC. GRK5 plays oncogenic roles in GBM, prostate, pancreas, renal cell, non-small-cell lung, and breast cancers (Kim et al., 2012; Gambardella et al., 2016; Komolov et al., 2017; Jiang et al., 2018; Yang et al., 2018; Alshabi et al., 2019; Zhao et al., 2019a; Zhao et al., 2019b; Lagman et al., 2019; Sommer et al., 2019). It is clear (Gambardella et al., 2016) that when GRK5 is localized at the plasma membrane, it often exerts an anti-tumoral effect, attenuating growth-associated signaling pathways through its ability to desensitize GPCR and non-GPCR receptors. However, when GRK5 moves to cytosol or nucleus, it often promotes tumor growth acting on nonreceptor substrates. Consistent with previous findings (Kim et al., 2012; Jiang et al., 2018; Alshabi et al., 2019; Zhao et al., 2019a), in the present study, our results showed that GRK5 was upregulated in paraffin-embedded and fresh EOC tissues, and it is located mainly at the nucleus, which suggested that GRK5 may play a candidate oncogenic role in EOC. Further, Kaplan–Meier survival analysis showed that GRK5 high expression was associated with shorter OS, but not RFS, which is consistent with the previous reports (Jiang et al., 2018; Zhao et al., 2019a) that GRK5 high-expression NSCLC or renal cell carcinoma patients had a worse OS rate than the low-expression patients. Furthermore, GRK5 expression was associated with the following clinicopathological parameters, such as: FIGO stage, intraperitoneal metastasis, intestinal metastasis, differentiation grade, and ascites with tumor cells, which showed that GRK5 high expression was closely related with the development and metastasis of EOC.

Similar to GRK5, ACTC1 is also a cardiac-related gene (Kondrashov et al., 2018), and both of them are the candidate interacting proteins of MYH10. ACTC1 encodes the cardiac form of actin (Kondrashov et al., 2018). Ohtaki et al. reported (Ohtaki et al., 2017) that ACTC1 served as a clinical marker to detect migration and poor prognosis in GBM patients. In addition, our results also demonstrated that ACTC1 was upregulated in paraffin-embedded and fresh EOC tissues compared with that in paratumor tissues, which suggested that ACTC1 played a candidate oncogenic role in EOC. This is consistent with the role of ACTC1 (Kim et al., 2019; da Rocha et al., 2019; Wanibuchi et al., 2018; Cheung et al., 2017; Li et al., 2017; Ohtaki et al., 2017; Zaravinos et al., 2011) in GBM, colon, prostate, oral squamous cell and breast cancers. Further, Kaplan–Meier survival analysis showed that ACTC1 high expression was closely associated with shorter OS and RFS, which is consistent with the previous study of ACTC1 in GBM. Furthermore, ACTC1 expression was associated with clinicopathological parameters of EOC, such as: FIGO stage, lymph node metastasis, intraperitoneal metastasis, intestinal metastasis, and vital status, which showed that ACTC1 was also closely associated with the development and metastasis of EOC.

Importantly, ACTC1 and GRK5 are both cardiac-related genes. In this study, our results showed that ACTC1 mRNA and protein expression were both positively correlated with GRK5 mRNA and protein expression, and Kaplan–Meier survival analysis showed that GRK5+/ACTC1+ was closely associated with poor OS and RFS. Multivariate Cox regression analysis showed that GRK5+/ACTC1+ co-expression was an independent prognostic factor rather than GRK5 or ACTC1 alone. Moreover, GRK5-/ACTC1+ patients had a worse survival than GRK5+/ACTC1- patients, which suggested that ACTC1 was more closely correlated with survival than GRK5. In addition, GRK5/ACTC1 co-expression was associated with the following factors: histology, FIGO stage, lymph node metastasis, intraperitoneal metastasis, intestinal metastasis, vital status, differentiation grade, and ascites with tumor cells. All of these results suggested that the combination of GRK5 and ACTC1 could predict the development, progression, metastasis, and prognosis of EOC more precisely. In future, we need more *in vivo* (such as the subcutaneous xenograft tumor studies and the lung xenograft tumor studies, and so on) and *in vitro* research to demonstrate its role and molecular mechanism in development, progression, metastasis, and prognosis of EOC.

## Conclusion

Taken together, the results of the present study suggest that GRK5 and ACTC1 are both upregulated in EOC, and GRK5+/ACTC1+ co-expression could predict the development, metastasis, and prognosis of EOC. The co-expression of GRK5+/ACTC1+ can be recommended as prognostic-predicting biomarkers in EOC, and it may provide an important value in the clinical therapy and supervision of EOC.

## Data Availability

The original contributions presented in the study are included in the article/Supplementary Material, further inquiries can be directed to the corresponding author.
